# Advanced Dermatofibrosarcoma Protuberans Treatment With Imatinib: Experience From a Dedicated Sarcoma Medical Oncology Clinic in India

**DOI:** 10.1200/JGO.18.00007

**Published:** 2018-07-26

**Authors:** Sameer Rastogi, Ekta Dhamija, Adarsh Barwad, Aditi Aggarwal, Atul Sharma, Rambha Panday

**Affiliations:** **Sameer Rastogi**, **Ekta Dhamija**, **Adarsh Barwad**, **Atul Sharma**, and **Rambha Panday**, All India Institute of Medical Sciences; and **Aditi Aggarwal**, Maulana Azad Medical College, New Delhi, India.

## Abstract

**Purpose:**

Advanced dermatofibrosarcoma protuberans (DFSP) is an exceptionally uncommon disease with scarce literature, especially from developing countries. Molecular testing is unfortunately not available in India, and expert diagnosis by a sarcoma pathologist is available only in tertiary care centers.

**Materials and Methods:**

We retrospectively analyzed consecutive patients with inoperable DFSP (on the basis of expert histopathology only) who presented to our sarcoma medical oncology clinic from January 2016 to July 2017.

**Results:**

There were a total of seven patients, with median age of 35 years, predominantly males (85.7%). Fibrosarcomatous variant and metastatic disease were present in six (85.7%) patients. Partial response rates were 71.4%, and overall disease control was 85.7%. Median progression-free survival was 14 months.

**Conclusion:**

DFSP diagnosis on the basis of expert histopathology in the absence of translocation can help out in targeted therapy–based treatment until translocation testing becomes available. The fibrosarcomatous variant has poor outcome, and further research is needed to help this group of patients.

## INTRODUCTION

Dermatofibrosarcoma protuberans (DFSP) is an exceedingly rare subtype of soft tissue sarcoma, constituting of 1% of all sarcomas. Behaviorally, DFSP rarely metastasizes (occurring in 5% of all cases), and the literature on metastatic DFSP remains conspicuously sparse.^1^ Histologically, DFSP can be classified as either classic or fibrosarcomatous (FS) variant. FS variant is characterized by more spindle cells, greater number of nuclei, and increased mitotic rate; unlike classic variant, immunohistochemically, CD34 expression is weak.^2^

Elucidation of molecular mechanisms of DFSP has resulted in the development of targeted therapy directed toward platelet-derived growth factor β (PDGFβ) in advanced disease. Tumors of DFSP are characterized by pathologic chromosomal rearrangement that fuses the COL1A1 promoter gene of chromosome 17 to the PDGFβ gene of chromosome 22.^3^ Over-activation of PDGFβ receptor tyrosine kinase leads to cellular proliferation and tumor formation. Imatinib, a small molecular adenosine triphosphate analog, acts by competitively inhibiting the adenosine triphosphate–binding site of the PDGFβ receptor tyrosine kinase in DFSP and thus causing the downregulation of kinase activity leading to growth inhibition and apoptosis.^4,5^

Although it has been known that treatment with imatinib has been used on this tumor, research publications about its use are limited, possibly because of the rarity of DFSP. . After few case reports supporting the clinical use of imatinib,^6,7^ McArthur et al^8^ published the clinical and radiologic outcomes (Imatinib Target Exploration Consortium Study, B2225) in advanced DFSP (N = 10; locally advanced, n = 8; metastatic, n = 2) using imatinib 800 mg per day and found a response rate of 90%. The largest group of prospective data for advanced DFSP came from the pooled analysis of two distinct phase II trials conducted by the European Organisation for Research and Treatment of Cancer (EORTC) and SWOG groups, consisting of 24 patients with locally advanced and metastatic DFSP receiving doses of imatinib ranging from 400 and 800 mg, respectively. The objective response rate was 46%, and median time to progression was 1.7 years.^9^ There was no difference between the different doses of imatinib in terms of overall response rates and progression-free survival (PFS). Recently, Rutkowski et al^10^ analyzed 31 patients with locally advanced/metastatic DFSP treated with imatinib for long-term outcomes and prognostic factors. Presence of metastatic disease and FS variant were associated with worst prognosis.^10^ Here, we report the experience of consecutive patients with metastatic/unresectable DFSP presenting in the last 2 years to our sarcoma medical oncology clinic.

## MATERIALS AND METHODS

This is a retrospective study evaluating patients with locally advanced/metastatic DFSP who were registered in a sarcoma medical oncology clinic between January 2016 and July 2017, and followed up until November 2017. The pathology of all the cases was reviewed by a sarcoma pathologist, and all cases were reviewed in a multidisciplinary clinic.

The dose of imatinib used in the clinic depended on the physician’s discretion and the response and tolerance of the patient. Data were studied through hospital records, including the age, sex, site, metastatic lesions, histopathology, dose of imatinib, response rate, and outcomes. The statistical analysis was done through SPSS 23 (SPSS, Chicago, IL). Nominal data are provided as number (%) and continuous data as median (range). PFS was calculated from the date of random assignment to the first date of documented progressive disease or the date of death from any cause.

## RESULTS

**Fig 1:**
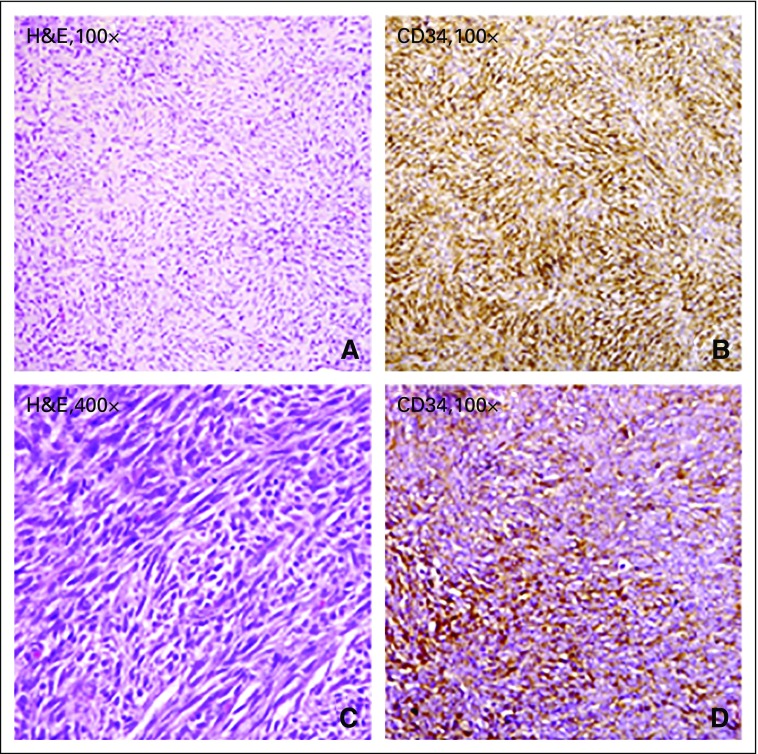
(A) Uniform population of spindled tumor cells arranged in a distinct, monotonous, storiform pattern around an inconspicuous vasculature. There is minimal nuclear pleomorphism and only low mitotic activity. (B) Immunohistochemistry performed for CD34 showing diffuse immunopositivity in the tumor cells. (C) Fibrosarcomatous dermatofibrosarcoma protuberans, where the tumor cells are arranged in fascicular architecture and herringbone pattern (loss of storiform pattern), with moderate nuclear pleomorphism and increased mitotic activity. (D) Immunohistochemistry performed for CD34 showing loss of CD34 immunostaining in a case of fibrosarcomatous transformation. H&E, hematoxylin and eosin.

**Fig 2:**
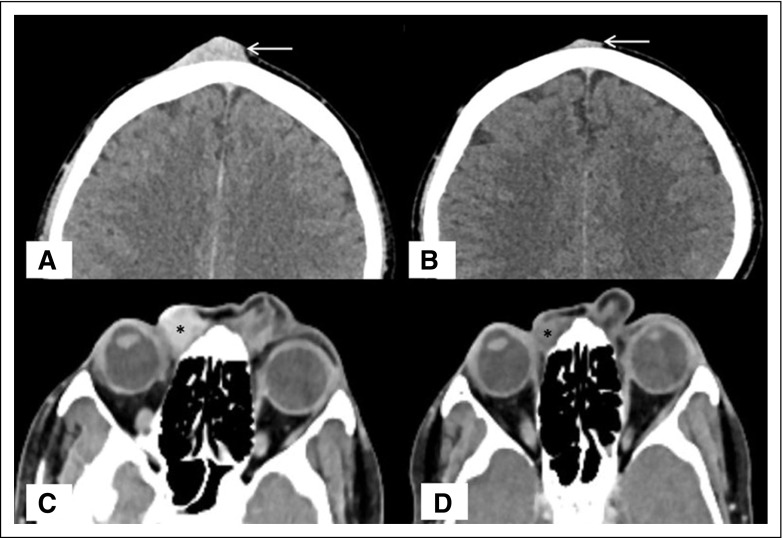
Dermatofibrosarcoma protuberans, partial response. (A, C) Baseline scan showed presence of homogeneously enhancing soft tissue in frontal region in midline (arrow in A) and along the medial canthus of right eye in extraconal compartment (asterisk in C) with no associated osseous destruction. (B, D) After initiation of treatment (after 4-month interval), both lesions showed significant reduction in size (arrow in B and asterisk in D), which remained stable until the last follow-up.

**Fig 3:**
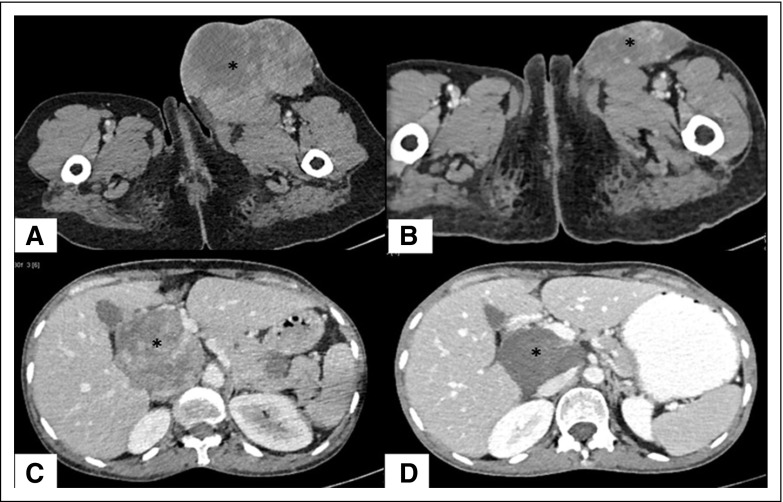
Dermatofibrosarcoma protuberans, fibrosarcomatous variant. The patient presented with multiple soft tissue as well as intra-abdominal and lung deposits, which were histopathologically proven to be fibrosarcomatous dermatofibrosarcoma protuberans with lung and bone metastases (not shown). (A) The baseline axial computed tomography image shows a large heterogeneously enhancing lesion in the left inguinal region and anteromedial aspect of the upper thigh (asterisk) infiltrating overlying skin and underlying muscles. (B) After 5 months of imatinib, the lesion showed marked reduction in size (asterisk), suggesting partial response to therapy. (C) Another lesion seen in the portocaval location (asterisk) on pretreatment computed tomography scan also showed reduction in size and enhancement (asterisk in D) indicating partial response to imatinib. The other lesions in bone and lungs also showed partial response (not shown) as reduction in size of soft tissue component

**Fig 4:**
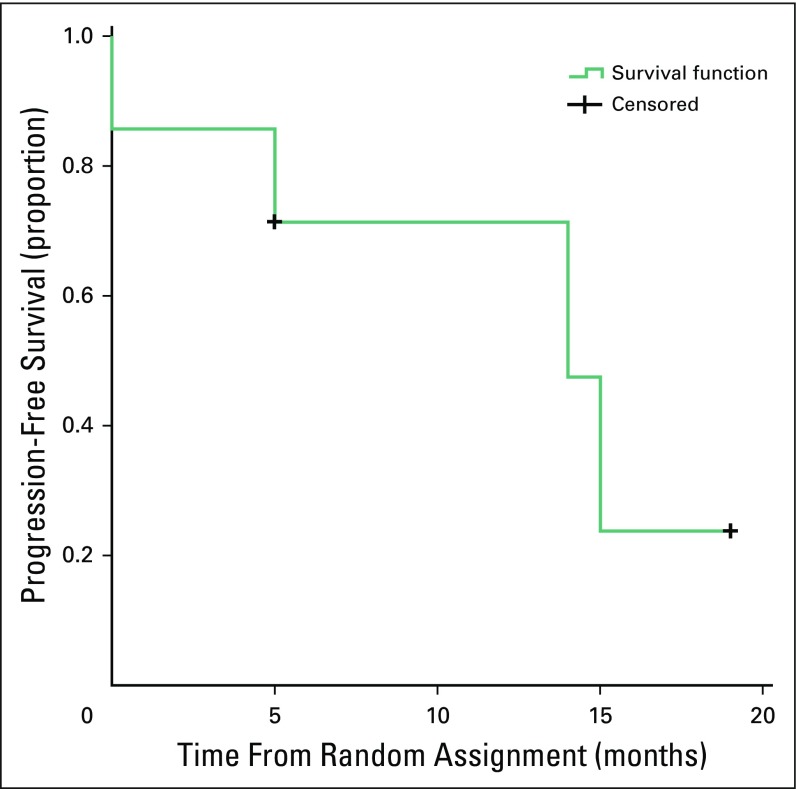
Progression-free survival in months.

A total of seven patients with the diagnosis of metastatic (n = 6) or locally advanced (n = 1) DFSP were referred to the sarcoma medical oncology clinic during this time. Median age of the patients was 35 years (age range, 19 to 54 years). The majority of the patients were men (n = 6; 85.7%). The locations of the primary lesion are listed in Table 1 (one forehead and eyelid; one nape of neck; five trunk). The median time from baseline diagnosis to development of metastasis/unresectablity was 42 months (range, 24 to 120 months). Of seven patients at the time of presentation to our clinic, six patients had FS transformation and one patient had classic DFSP (Fig 1). Patient characteristics are summarized in Table 1. Best response was partial response in five patients (responses for patients 1 and 7 are shown in Figs 2 and 3, respectively), stable disease in one patient, and progressive disease in one patient. Table 2 lists treatment characteristics, including dose of imatinib, response, and current status. Of all patients with metastatic disease (n = 6), the most common site of metastasis was the lung (83%), followed by soft tissue (66.6%) and bones (33%). The median number of metastatic sites was three (range, one to five), suggesting high burden of disease. Of those patients who started chemotherapy, four patients have experienced progression to date (Table 3). Median PFS in our patients was 14 months (Fig 4). One patient was lost to follow-up, and the rest were followed until the last date of follow-up.

**Table 1:**
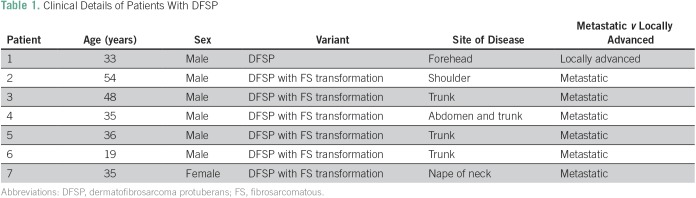
Clinical Details of Patients With DFSP

**Table 2:**
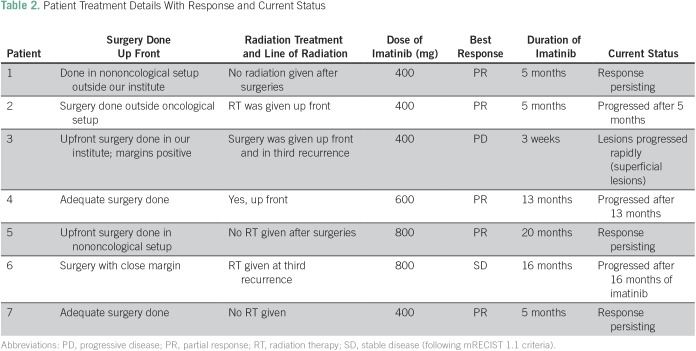
Patient Treatment Details With Response and Current Status

**Table 3:**
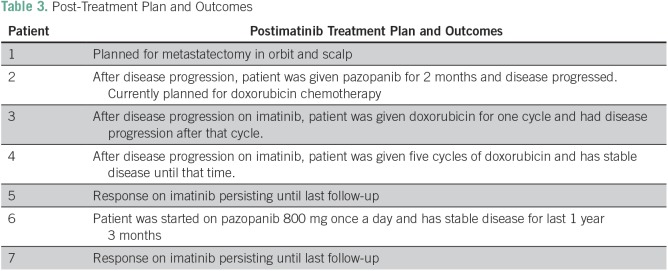
Post-Treatment Plan and Outcomes

## DISCUSSION

The role of imatinib is well established in chronic myeloid leukemia and GI tumors.^11^ The data regarding the outcome of advanced DFSP treated with imatinib are sparse, and in the developing world they are limited to occasional case reports only.^12,13^ This could be because of a lack of expert histopathology, lack of translocation testing, and absence of dedicated sarcoma clinics and multidisciplinary teams. This is the first series from India with consecutive patients with advanced DFSP from the sarcoma medical oncology unit of a tertiary care institute.

Our patients presented a decade earlier than the SWOG/EORTC pooled analysis data and B2225 study, and this could be attributed to the younger population structure of Indian patients.^8,9^ There was male predominance in our series, as has been shown in the previous SWOG/EORTC pooled analysis. However, in the study by McArthur et al^8^ (B2225 study), there was an equal number of male and female patients. The time from baseline diagnosis to metastasis/unresectability was 42 months, which was 34 months in the SWOG/EORTC pooled analysis.^9^ The trunk was the most common primary site, followed by the head and neck, similar to the SWOG/EORTC pooled analysis; in the B2225 study, the head and neck was the most common site (30%). We had six out of seven (85.7%) patients with FS transformation, in contrast to the other studies where the FS variant was 43% to 52%.^9,10^ The response rate in our series was 71%. Response rates in various studies have been shown to be between 46% and 90%, with FS variant having the shortest lasting responses.^8-10^ PFS has varied in the literature in different studies. For example, in the pooled analysis by SWOG/EORTC, 1-year PFS was 57.18%, whereas in another retrospective analysis the 5-year PFS was 58%, although the percentage of FS variant and patients with metastatic disease was almost the same in both studies.^9,10^ Shorter PFS of 14 months in our study could be explained by the predominance of FS variant and metastatic disease along with high disease burden.

Limitations of this study are that it was a small, retrospective study with limited follow-up, which is frequently the case with rare tumors. We have not done mutation testing in our cohort, but we believe that partial response/stable disease while receiving imatinib reinforces the diagnosis in the setting of a tertiary care bone and soft tissue–specific pathologist. This is the first case series to our knowledge from the developing world solely on the basis of the histopathology, and the results are encouraging. Until we have molecular techniques available, good histopathology reporting and multidisciplinary management can be the cornerstone of the treatment of this rare disease.

## References

[1] Bowne WB, Antonescu CR, Leung DH (2000). Dermatofibrosarcoma protuberans: A clinicopathologic analysis of patients treated and followed at a single institution. Cancer.

[2] Achouri L, Triki A, Bouzaiene H (2016). Transformed dermatofibrosarcoma protuberans: A series of nine cases and literature review. Journal of Dermatology and Dermatological Surgery.

[3] Sandberg AA, Bridge JA (2003). Updates on the cytogenetics and molecular genetics of bone and soft tissue tumors. Dermatofibrosarcoma protuberans and giant cell fibroblastoma. Cancer Genet Cytogenet.

[4] Simon MP, Pedeutour F, Sirvent N (1997). Deregulation of the platelet-derived growth factor β-chain gene via fusion with collagen gene COL1A1 in dermatofibrosarcoma protuberans and giant-cell fibroblastoma. Nat Genet.

[5] Sjöblom T, Shimizu A, O’Brien KP (2001). Growth inhibition of dermatofibrosarcoma protuberans tumors by the platelet-derived growth factor receptor antagonist STI571 through induction of apoptosis. Cancer Res.

[6] Maki RG, Awan RA, Dixon RH (2002). Differential sensitivity to imatinib of 2 patients with metastatic sarcoma arising from dermatofibrosarcoma protuberans. Int J Cancer.

[7] Gronchi A, Stacchiotti S, Pedeutour F (2008). Response to imatinib mesylate (IM) in fibrosarcoma (FS) arising in dermatofibrosarcoma protuberans (DFSP). J Clin Oncol.

[8] McArthur GA, Demetri GD, van Oosterom A (2005). Molecular and clinical analysis of locally advanced dermatofibrosarcoma protuberans treated with imatinib: Imatinib Target Exploration Consortium Study B2225. J Clin Oncol.

[9] Rutkowski P, Van Glabbeke M, Rankin CJ (2010). Imatinib mesylate in advanced dermatofibrosarcoma protuberans: Pooled analysis of two phase II clinical trials. J Clin Oncol.

[10] Rutkowski P, Klimczak A, Ługowska I (2017). Long-term results of treatment of advanced dermatofibrosarcoma protuberans (DFSP) with imatinib mesylate: The impact of fibrosarcomatous transformation. Eur J Surg Oncol.

[11] Iqbal N, Iqbal N (2014). Imatinib: A breakthrough of targeted therapy in cancer. Chemother Res Pract.

[12] Mahajan BB, Sumir K, Singla M (2015). Metastatic dermatofibrosarcoma protuberans: A rare case report from North India. J Cancer Res Ther.

[13] Ta RK, Banerjee SN (2015). Dermatofibrosarcoma protuberans: A rare presentation with lung and abdominal metastasis. Medical Journal of Dr DY Patil Vidyapeeth.

